# COVID-19 and Cardiovascular Surgery. Do We Know What We Are Dealing With?

**DOI:** 10.21470/1678-9741-2021-0962

**Published:** 2021

**Authors:** Rui M. S. Almeida, Mateo Marin-Cuartas, Ovidio A. Garcia-Villarreal, Victor Dayan

**Affiliations:** 1 Centro Universitário Fundação Assis Gurgacz, Cascavel, Paraná, Brazil.; 2 University Department of Cardiac Surgery, Leipzig Heart Center, Leipzig, Germany.; 3 Mexican College of Cardiovascular and Thoracic Surgery, Mexico City, Mexico.; 4 Centro Cardiovascular Universitario, Montevideo, Uruguay.

The coronavirus disease 2019, also known as the COVID-19 pandemic, has been a source of immense strain, heavily battering the healthcare systems and financial stability all over the world and imposing a sudden and critical disruption of the established triage, testing services, and referral and counter-referral pathways.

Patients with cardiovascular diseases (CVD) and in need of cardiovascular surgery have been badly hit. Cardiovascular risk factors are associated with COVID-19 adverse outcomes, and evidence suggests that adults with CVD and COVID-19 are at a higher risk of in-hospital mortality.

In this issue of the Brazilian Journal of Cardiovascular Surgery, the report of the Brazilian nationwide experience of COVID-19 complicating the perioperative period of cardiovascular surgery adds to make clear this relationship and reveals the toll taken on these patients, with an overall mortality rate topping 24%. The finding of the temporal correlation - the longer the timespan between the positive reverse transcription-polymerase chain reaction (RT-PCR) for severe acute respiratory syndrome coronavirus 2 (SARS-CoV-2) and the time of operation the better the outcomes - provides valuable information for safer management of patients at risk. However, while postponing elective operations might be safer in low-risk patients, in high-risk patients the longer delays have an impact on outcomes, leading to worse prognoses^[1]^.

Decisions to defer surgery should be customized for each patient, the likely benefits of delaying surgery following positive RT-PCR for SARS-CoV-2 must be balanced against the potential risks. The findings of the Brazilian study are in line with a large international study, which showed that keeping surgery on hold for at least seven weeks after a positive coronavirus test was associated with lower mortality risk compared with no delay^[2]^.

Worldwide, a great number of elective cardiac surgeries have been postponed only being performed in urgent and emergency conditions. In the United Kingdom, the volume of surgical activity was reduced by 33.6% in 2020, resulting in more than 1.5 million canceled operations, and this deficit is believed to continue to grow in 2021^[3]^.

Gaudino et al. quantified the experience and changes implemented in response to the COVID-19 pandemic across cardiac surgery centers participating in an international research consortium. The median reduction in cardiac surgery case volume worldwide was 50% to 75%, correlating with the number of local COVID-19 cases. Most centers restricted cardiac surgery activity to urgent and emergency cases; 5% had canceled all cases, including emergencies. South American centers reported fewer reductions in cardiac surgery case volume, with Asian centers being the more affected^[4]^.

In Brazil, it is estimated that < 40,000 patients underwent heart operations in 2020, a drop of more than half in relation to a figure of approximately 100,000 operations annually performed in the country. The situation was aggravated by a shortage in the supply of essential materials for these surgeries, like heart valve prosthesis, cardiopulmonary bypass sets, and cannulas, used in coronary artery bypass surgery and valve replacement, correction of congenital heart defects, aortic aneurysm repairs, and others^[5,6]^.

An increase in the number of cases operated on as urgent/emergency during the pandemic is a reflection of the disease severity, either as a consequence of the patient's delayed presentation to the hospital or directly from COVID-19 complications^[7]^.

A significant proportion of healthcare resources has been diverted to the care of COVID-19-affected patients. The disruption to surgery in Brazil and Latin America during the pandemic, with the sharp reduction of the volume of surgical activity and the number of canceled surgical procedures, will continue to affect millions of patients for many years, given the gravity of the growing elective backlog and the restrained health budget. Therefore, the overall impact of the pandemic is likely to become evident over the coming years, and drastic measures will be necessary to mitigate these backlogs.

Additionally, this provision has, directly and indirectly, affected the learning curve of residents of the surgical area, as is the case with cardiovascular surgery evidencing a reduction in surgical procedures (elective vs. emergency), leading to a decrease in the hours of surgical training, progressively replaced by support hours in specific areas for COVID-19, like intensive care units and emergency rooms. A variety of technological alternatives has been suggested to try to compensate for the lack of traditional academic training in medical residency towards a virtual hybrid model^[8,9]^.

The faster restoration of healthcare pathways with a major reorganization of the services and the commitment of additional budget by the public and private sources for surgery for the years to come would help bring the waiting list back to more workable levels.

The COVID-19 pandemic leaves a lesson, with a need by authorities for a more rapid response to the constantly changing scenarios. The spectrum of successive waves with a surge in the new cases leading to excess hospitalizations and deaths from COVID-19 spreads the fear of the worst is not over. The emergence of a highly transmissible variant of SARS-CoV-2 circulating right now in Europe and Asia, coupled with waning immunity and relaxation of public policies and infection prevention measures, resulting in national lockdown and further cancellations of operations, is a clear reminder of the uncertain times still ahead, with patients expected to face even longer waits and threats. A notice for not letting our guard down anytime soon. In this way, Ibarra & Duarte have proposed multiple evidence strategies to resume these services in Latin America and reverse the backlog, restraining the risks of contamination both from and to the patient^[10]^.

In sum, evidence conveyed by the Brazilian nationwide experience on managing patients with perioperative COVID-19 showing a higher rate of morbidity and mortality should be part of our clinical decision-making for selecting the best care strategy for our patients. By following strict safety guidelines, careful and planned strategies in RT-PCR-positive patients help improve risks of perioperative complications and death.
